# Semi-supervised nuclei segmentation based on multi-edge features fusion attention network

**DOI:** 10.1371/journal.pone.0286161

**Published:** 2023-05-25

**Authors:** Huachang Li, Jing Zhong, Liyan Lin, Yanping Chen, Peng Shi

**Affiliations:** 1 College of Computer and Cyber Security, Fujian Normal University, Fuzhou, Fujian, China; 2 Department of Radiology, Clinical Oncology School of Fujian Medical University, Fujian Cancer Hospital, Fuzhou, Fujian, China; 3 Department of Pathology, Clinical Oncology School of Fujian Medical University, Fujian Cancer Hospital, Fuzhou, Fujian, China; 4 Digit Fujian Internet-of-Things Laboratory of Environmental Monitoring, Fujian Normal University, Fuzhou, Fujian, China; The University of Lahore, PAKISTAN

## Abstract

The morphology of the nuclei represents most of the clinical pathological information, and nuclei segmentation is a vital step in current automated histopathological image analysis. Supervised machine learning-based segmentation models have already achieved outstanding performance with sufficiently precise human annotations. Nevertheless, outlining such labels on numerous nuclei is extremely professional needing and time consuming. Automatic nuclei segmentation with minimal manual interventions is highly needed to promote the effectiveness of clinical pathological researches. Semi-supervised learning greatly reduces the dependence on labeled samples while ensuring sufficient accuracy. In this paper, we propose a Multi-Edge Feature Fusion Attention Network (MEFFA-Net) with three feature inputs including image, pseudo-mask and edge, which enhances its learning ability by considering multiple features. Only a few labeled nuclei boundaries are used to train annotations on the remaining mostly unlabeled data. The MEFFA-Net creates more precise boundary masks for nucleus segmentation based on pseudo-masks, which greatly reduces the dependence on manual labeling. The MEFFA-Block focuses on the nuclei outline and selects features conducive to segment, making full use of the multiple features in segmentation. Experimental results on public multi-organ databases including MoNuSeg, CPM-17 and CoNSeP show that the proposed model has the mean IoU segmentation evaluations of 0.706, 0.751, and 0.722, respectively. The model also achieves better results than some cutting-edge methods while the labeling work is reduced to 1/8 of common supervised strategies. Our method provides a more efficient and accurate basis for nuclei segmentations and further quantifications in pathological researches.

## Introduction

Over the decades, computational pathology has been one of the hotspots in biological and clinical researches [1]. As the basic unit of pathologic diagnosis, the nuclei/cells microenvironment delivers massive information [2]. With the statistical features change of normal cells after cancerization, nuclei numbers, size, morphology, and other indicators can be used for auxiliary diagnosis [3]. The accurate segmentation of nuclei from the stained pathological image is essential in the research of pathological diagnosis, which is typically done by radiologists who spend significant time on manual segmentation. However, due to the high density of whole slide images, it is impractical to segment millions of nuclei manually, which makes it valuable to construct an automatic model for nuclei segmentation. Owing to the diversity of uncertain cell boundaries after staining, automated nuclei segmentation of pathological images remains challenging in clinical pathology analysis. In the early stages, image-processing-based nuclei segmentation methods were commonly used including threshold determination [4], contour evolution model construction [5], and seed point marking [6]. Afterwards, machine-learning-based methods were developed by considering segmentation as pixel or small patch-based classification, such as K-means clustering [7], fuzzy C-means clustering [8] and Support Vector Machine (SVM) [9]. Although classic image-processing-based and machine learning-based methods have made strides in medical image segmentation, it is still difficult to include high representative features and lacks neighborhood receptive fields. In recent years, the methods based on deep learning developed rapidly and have been applied in medical image segmentation. Convolutional Neural Network (CNN) [10] and CNN-based models have achieved outstanding performance in medical image segmentation while such methods mainly rely on manual labeling of sufficient sample images and can’t meet efficiency requirements in clinic. Accurate segmentation of individual primitives based on effective feature extraction is a challenging problem for pathological images [11] due to issues such as sparse chromatin, background mottle interference, and appearance diversity. In recent years, image segmentation methods based on the neural network have made significant progress, and it is gradually being applied in pathological image segmentation. The method of the neural network approaches any algorithm theoretically, but such a method can be challenging to train and get satisfactory results. For tasks in specific areas, it need to find a suitable network structure and adjust the input. In addition, considering that the existing models have achieved outstanding performance under the supervision of dense annotations, reducing training samples while maintaining a reasonable performance is still an open problem [12].

In this paper, a novel MEFFA-net with pseudo-mask and edge-aware blocks is brought forward for more accurate and efficient nuclei segmentation, which also includes a semi-supervised pseudo-mask augmentation strategy. The MEFFA-Net takes the rough pseudo-mask generated by pre-trained U-Net and the edge features extracted by Canny operator as two additional inputs, and takes the nuclear edge detection as an additional auxiliary task to improve the accuracy of the segmentation task. The selection and rational use of depth features are performed by MEFFA blocks. A shortcut is added between the input and the decoder output to make the network focus more on the details of the nucleus, which is the difference between the pseudo-mask generated by U-Net and manual annotation. This method has the function of making the coarse pseudo-mask more accurate, and it can be used to design a semi-supervised pseudo-mask enhancement strategy. The rest of the paper is organized as follows. In Section 2, literature review is discussed. In Section 3, detailed model is described, in which the loss function and the semi-supervised pseudo-mask augmentation strategy are defined. In Section 4, public pathological image datasets from multi-organs are introduced as baselines of the experiments. Comparative experiment results are presented in Section 5 to show the achieved improvements which cover both quantitative and qualitative evaluations on the multiple datasets. Then, the ablation experiment results are shown as well. Finally, conclusions and discussions on the proposed method are conducted. Our contributions are summarized as follows: First, we propose a novel deep-learning-based network for pathological image segmentation (MEFFA-Net) and design a Multi-Edge Feature Fusion Attention module for fusing edge features from images, image pseudo-masks, and image edge detection results. The above improvements allow the network to learn more information from multiple resources for the nuclei segmentation of pathological images. Second, we obtain more reliable pseudo-masks by using MEFFA-Net which takes coarse pseudo-masks as input and outputs more precise segmentation masks. Therefore, we solve the cost problem of the manual outlining pathological images to a certain extent. Third, extensive comparison and ablation experiments have been performed on the MoNuSeg, CPM-17, and CoNSeP public pathology image datasets. The results show that MEFFA-Net provides performance improvements consistently and significantly.

## Literature review

In terms of pathological image segmentation, the segmentation model based on CNN prediction is one of the more successful techniques. Yang et al. [13] constructed a CNN2 model composed of two convolution-pooling layer pairs and two fully connected layers for histopathological nuclei images and realized end-to-end model training. On this basis, Kumar et al. [14] proposed a deeper CNN3 model to achieve the segmentation of a wider range of pathological images. Yi et al. [15] adopted a unified Fully Convolutional Network (FCN) framework to model detection and segmentation tasks. As the improvement of FCN, the encoder-decoder model is widely used in computer vision and artificial intelligence. Among the existing encoder-decoder models, U-Net [16] is one of the most widely used techniques in pathological image segmentation, which solves the segmentation problem of complex neuron structures. Further, as an extension of U-Net, V-Net [17] realizes end-to-end 3D deep supervised learning, and by introducing a residual module and Dice loss function, it avoids the situation of an unbalanced number of front and background voxel categories. Some other encoder-decoder models have also been applied to the semantic segmentation of pathological images. For example, Chen et al. [18] and Fakhry et al. [19] put forward a deep contextual segmentation network and a residual deconvolution segmentation network for brain neuron images, respectively. Saha et al. [20] raised the Her2Net segmentation network for human epidermal growth factor receptor-2 (HER2) breast cancer cell images. Xie et al. [21] posed a deep segmentation correction network for colorectal adenocarcinoma images. These encoder-decoder models have a great segmentation effect in pathological images but still lack full utilization of the underlying information of pathological images, resulting in rough segmentation results. Subsequently, the researchers introduce an attention mechanism [22, 23], which evaluates the importance of different location features by assigning weights, and then models the context dependencies of local features. Furthermore, the attention mechanism is combined with the codec model and applied to the semantic segmentation of pathological images. Classic examples include the attention nerve cell segmentation model, the channel and spatial attention segmentation model, etc. Simultaneously, since the emergence of the GAN [24] in 2014, the research literature on its application in pathology has continued to emerge. With the introduction of CGAN [25], adversarial segmentation methods learn a context-aware structured network loss by increasing the size of the receptive field, thereby solving the problem of cell segmentation under complex background structures. In addition, there are more and more image analysis methods to improve the input, which can provide more prior information for the model. For example, Qin et al. [26] proposed a multi-focus image fusion method based on sparse representation for the case of defocused images, which achieves the effect of defocused image deblurring by fusing multiple images. Xiong et al. [27] proposed a new single-stage 3D object detection network based on density clustering and graph neural network. The density clustering ball query optimizes the point cloud space divided by the original ball query to ensure that the key point set contains more detailed object features. Lu et al. [28] reconstructed better image results by adding low-dose CT images obtained by image post-processing based on learning sparse transform to prior information, and solved the problem of improving CT imaging quality while reducing radiation dose. In this paper, the idea of providing more prior information is combined with the actual needs of the nuclei segmentation task to solve the problem of nuclei segmentation. We use the pseudo-mask obtained by pre-training U-Net and the edge features obtained by Canny operator as prior information, and use MEFFA-Block to fuse and select features to obtain a more accurate nuclei segmentation mask. The application of neural network methods has made great progress in various fields.

## Methodology

To solve the practical problem of nuclei segmentation in pathological images, the pipeline of MEFFA-Net is put forward with a semi-supervised pseudo-mask augmentation strategy for training the network.

### Multi-edge features fusion attention network

Considering more features that are beneficial to segmentation, such as edge features from the edge and position features from the pseudo-mask, the network can be better trained based on the following detailed structure designing.

#### Network structure

Since the development of deep learning, researchers have been exploring the network structure that can better accomplish the specific task. For the task of nuclei segmentation, a MEFFA-Net is designed which is described as the following.

**Fig 1:**
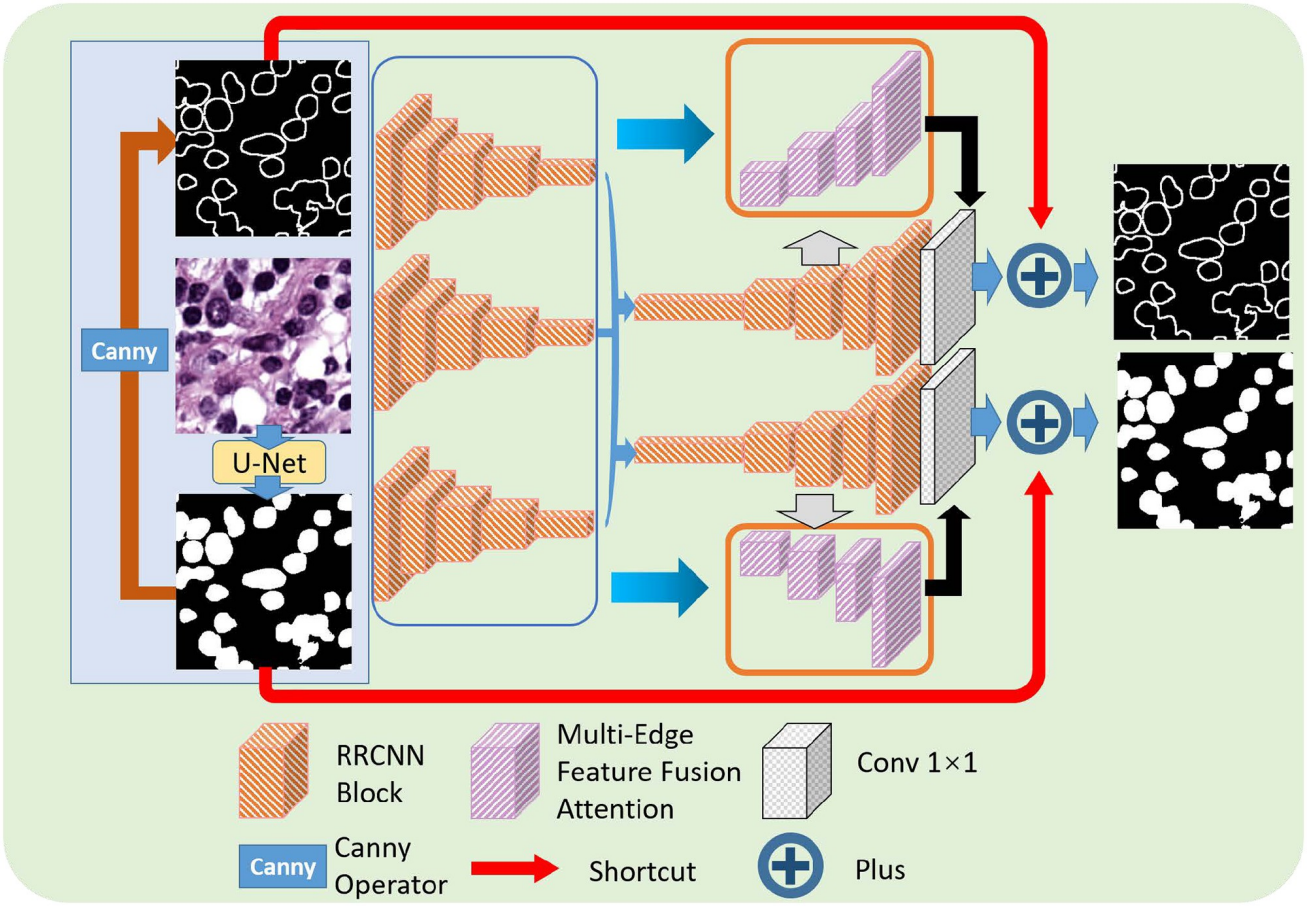
The structure of multi-edge features fusion attention network.

Fig 1 demonstrates the network structure of our proposed model. In our previous work [29], we used clustering-based methods to generate pseudo-masks and have made achievements. This round of work will explore the initial generation of pseudo-masks using U-Net. As illustrated in Fig 1, a trained U-Net is used to generate rough initial segmentation results which still suffer from under- over-segmentations but could offer pseudo-mask and its edge features extracted by the Canny operator. With the fusion of image, pseudo-mask and edge, better nuclei segmentation results can be created by the MEFFA-Net, providing a basis for further pathological diagnosis. As shown in Fig 1, three ways of features are extracted by the backbone network of the Recursive Residual U-Net [30] structure while an additional edge output, which helps the feature selection more related to the nuclei boundary. The orange block means Recursive Residual CNN Block [30], the purple block means the MEFFA-Block which will be described in the next section, and the grey block means 1×1 convolution.

Meanwhile, the features at each layer are passed to the MEFFA-Block for considering more information when creating more precise segmentation results. Focusing on multi-edge features of segmentation, the MEFFA-Net is well-trained in nuclei segmentation. Furthermore, a shortcut [31] connection between the rough segmentation and the output of the network is added to lead the network to learn detailed information. Similarly, another shortcut connection is introduced between the edge from the rough segmentation and the edge from the segmentation result.

#### Multi-edge features fusion attention block

**Fig 2:**
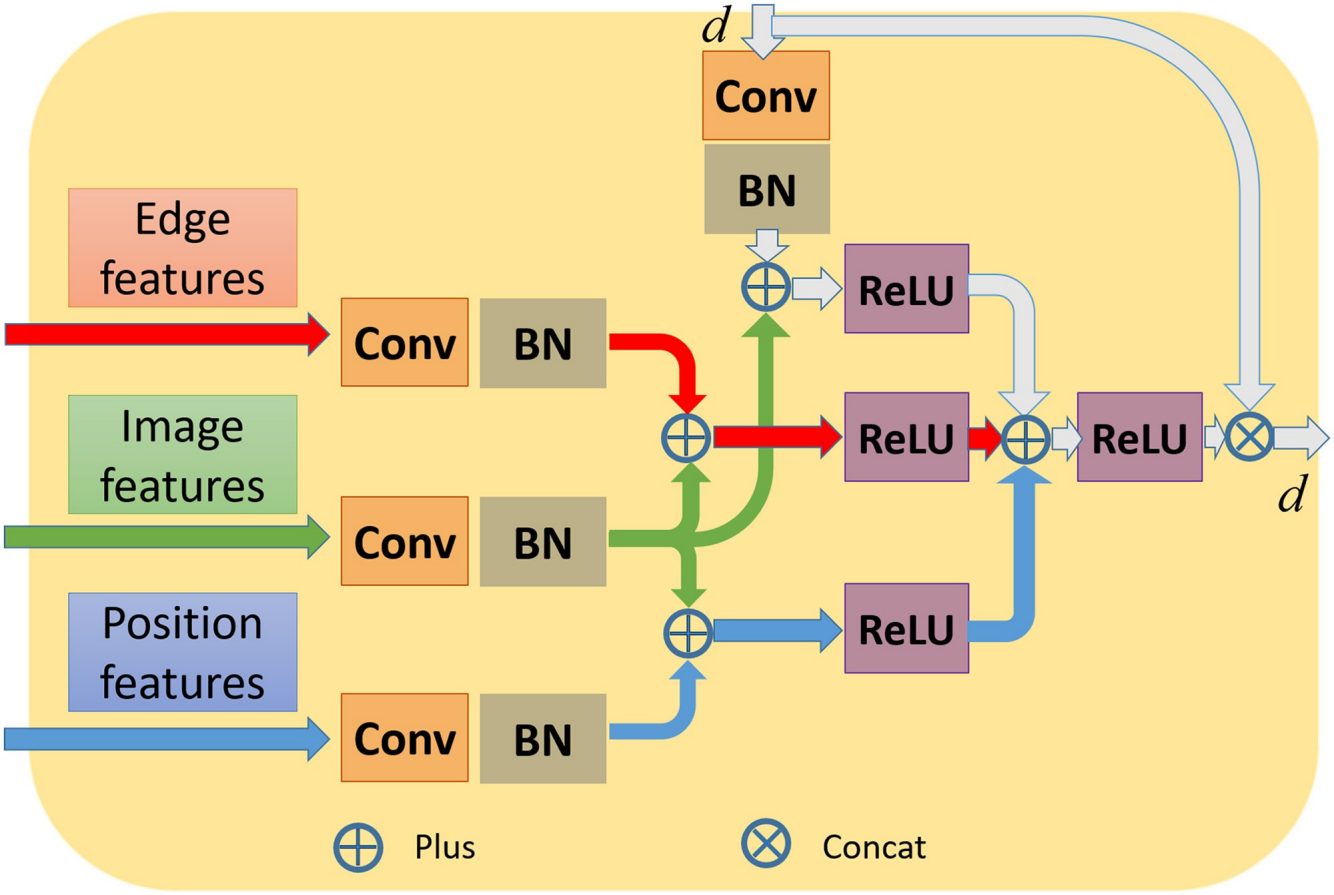
Details of the multi-edge features fusion attention block. The position features are from the pseudo-mask while the image features and the edge features are from image and edge, respectively.

Due to the lack of screening of multi-edge features, it is difficult for the network to learn better segmentation capabilities. Inspired by the idea of multi-head attention [32], the MEFFA-Block is put forward. As demonstrated in Fig 2, there is a three-way transmission of features at the same layer. The image features are separately plus the edge features, position features, and the output features of the previous encoder. The module’s output is the product of the sum of them and the output of the previous encoder. This attention module emphasizes the position and edge information in the task, which facilitates the network to produce more precise nuclei segmentation results and provides a more accurate basis for pathological diagnosis.

#### Loss function

The nuclei segmentation and edge detection are considered together in the loss function, and Dice [18] and Binary Cross Entropy losses are both considered in the total loss calculation.

$$\begin{array}{c} \Gamma_{\text{total}}=c_{1}\left(\Gamma_{seg\_Dice}+\Gamma_{seg\_BCE}\right)+c_{2}\left(\Gamma_{edge\_Dice}+\Gamma_{edge\_BCE}\right) \end{array}$$ (1)

As demonstrated in Table 1, the parameter *c*_1_ is in the range of 0–1 and *c*_2_ = 1 − *c*_1_. We use 1/32 of the labeled samples for the pre-experiment with 20 epochs. The pre-experiment is to confirm the value of the two parameters and only refers to IoU—which is one of the most classic image segmentation statistical results. According to the experimental results, when the weight ratio between each loss is equal, the network with excellent performance can be iterated out faster. As a result, the *c*_1_ and *c*_2_ are set as the constant of 1.

**Table 1:** Pre-experiment tuning of *c*_1_ and *c*_2_.

	*c*_1_ = 0.1	*c*_1_ = 0.2	*c*_1_ = 0.3	*c*_1_ = 0.4	*c*_1_ = 0.5	*c*_1_ = 0.6	*c*_1_ = 0.7	*c*_1_ = 0.8	*c*_1_ = 0.9
	*c*_2_ = 0.9	*c*_2_ = 0.7	*c*_2_ = 0.9	*c*_2_ = 0.6	*c*_2_ = 0.5	*c*_2_ = 0.4	*c*_2_ = 0.3	*c*_2_ = 0.2	*c*_2_ = 0.1
IoU	0.481	0.528	0.561	0.434	0.645	0.481	0.556	0.571	0.580

### Semi-supervised pseudo-mask augmentation strategy

To reduce the dependence on labeled samples while ensuring sufficient accuracy, a semi-supervised training strategy is introduced using the MEFFA-Net to enhance the pseudo-mask, which has the following detailed steps.

#### Classic semi-supervised learning based on pseudo-mask

The well-known semi-supervised teacher-student model [33] uses a few labeled samples to train a teacher model, then uses the teacher model to label a large batch of unlabeled samples, then the student model uses the pseudo-masks for learning. The U-Net is applied to the teacher-student model and the original pseudo-masks are generated.

As shown in Table 2, the labeled data is firstly used to train a U-Net, which can be used to annotate the unlabeled data. As it is able to get the F1 score of 0.76 on the MoNuSeg dataset, the annotation is reliable but needs improvement. Similar to classical semi-supervised teacher-student methods, labeled and unlabeled data are used to train another U-Net that takes charge of creating the original pseudo-mask.

**Table 2:** Details of generating the original pseudo-mask based on U-Net.

Step	Data	Processing	Output
1	Labeled data	Training	Mask
2	Unlabeled data	Label	Pseudo-mask
3	All data	Training	Mask & Pseudo-mask
4	Unlabeled data	Label	Pseudo-mask

Though the U-Net based semi-supervised method has reached great effect, it still can’t meet the precise requirements of pathological diagnosis for nuclei segmentation, while manual labeling is still in use and better segmentation methods need to be advanced.

#### Pseudo-mask augmentation strategy

The pseudo-mask augmentation strategy further improves the precision of nuclei segmentation with a small part of the labeled data and will be described in this section.

**Fig 3:**
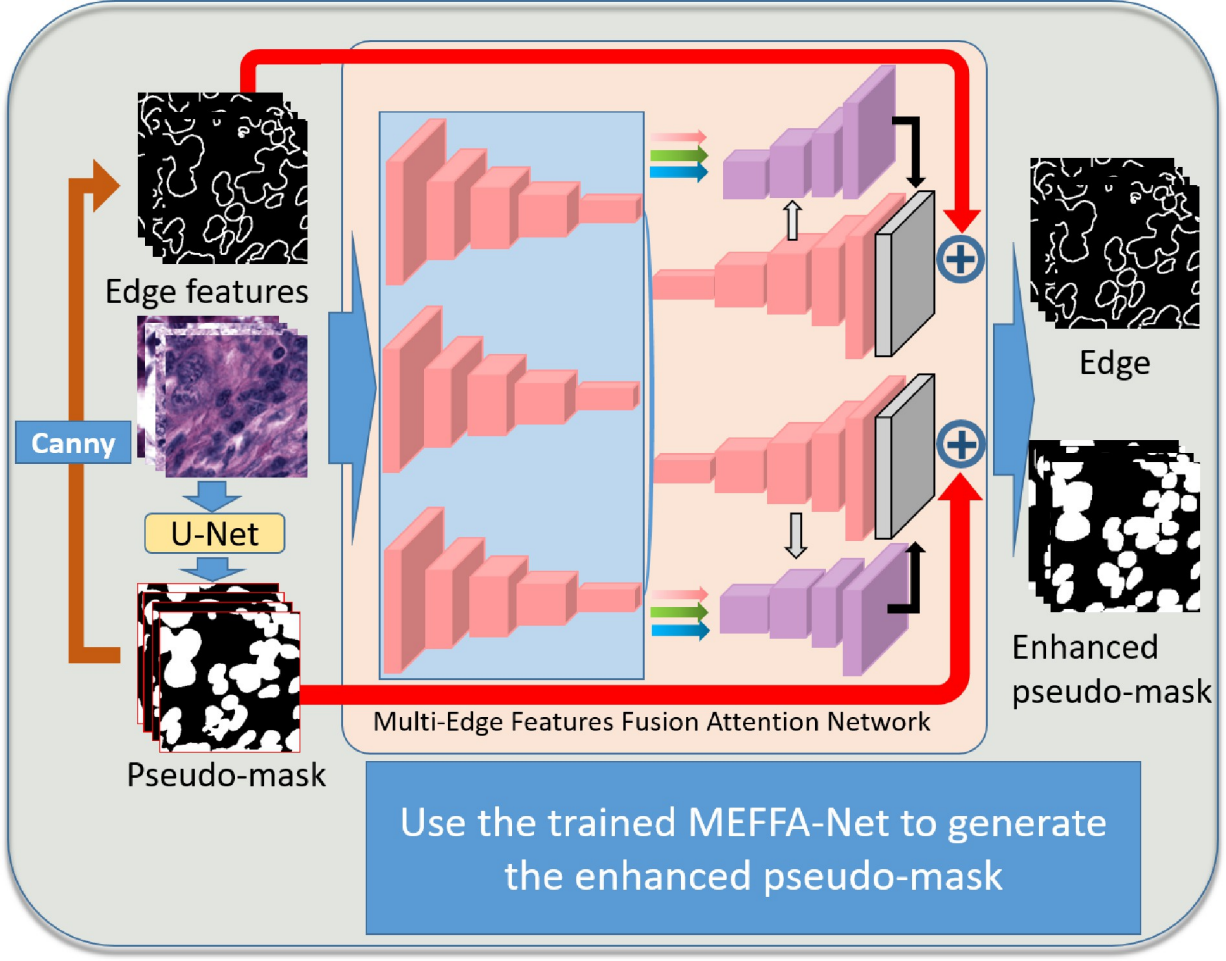
Details of generating the enhanced pseudo-mask.

As shown in Fig 3, based on the MEFFA-Net structure introduced earlier, a semi-supervised pseudo-label enhancement strategy can be implemented. First, A U-Net and a MEFFA-Net are trained using a few labeled data. Second, we use the trained U-Net to generate rough pseudo-masks, then use the canny operator to obtain edge feature maps from rough pseudo-masks. The above three are the input of the trained MEFFA-Net, and more accurate pseudo-masks than those generated by U-Net can be obtained at the network output.

**Fig 4:**
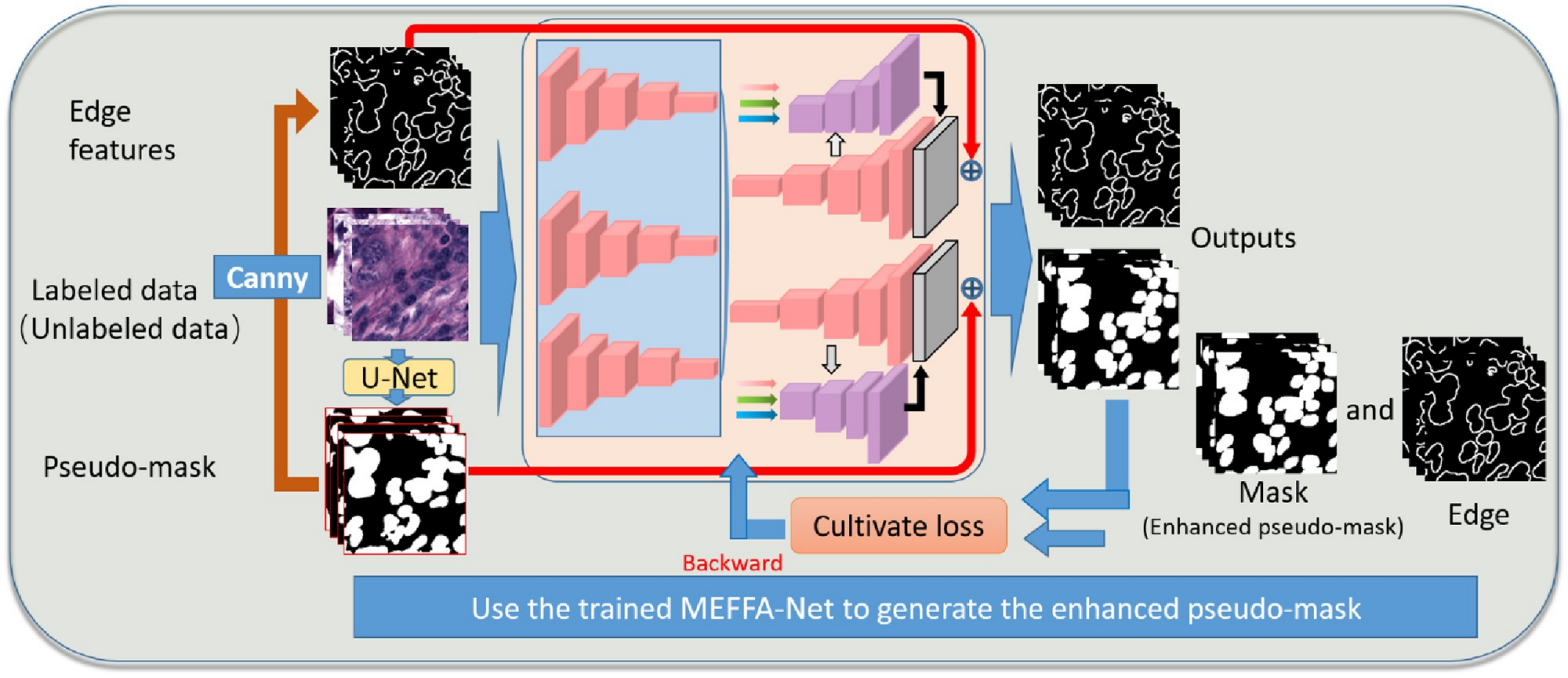
Details of the well-performed MEFFA-Net training using the semi-supervised pseudo-mask augmentation strategy.

Finally, as Fig 4 shows, all data is fully used to train a well-performed MEFFA-Net. For labeled data, its image, pseudo-mask, and edge are taken as inputs and the Dice and BCE losses between outputs and mask and edge backward to update the network’s parameters. For unlabeled data, its image, pseudo-mask, and edge from the pseudo-mask are also taken as inputs while the loss between outputs and enhanced pseudo-mask and edge from the enhanced pseudo-mask is adopted.

In the field of pathological image nuclei segmentation, the high labeling cost makes the research progress of automatic segmentation of pathological images slow. Scientists are focusing on semi-supervised and unsupervised learning, which have become hot spots. Our methods are exploring how to further develop the pathological image nuclei segmentation through more effective features input and model structure improvement. Further development of the semi-supervised nuclei segmentation technology is explored through the MEFFA-Net and the pseudo-mask augmentation strategy, making full use of the pathological nuclei images without a number of manual annotations by pathologists and being more in line with the needs of practical applications in pathology.

## Experiment settings

### Datasets and preprocessing

The proposed method is evaluated on three public nuclei segmentation datasets, details of which are presented in Table 3. The study was performed on the public released databases with no personal information provided.

**Table 3:** Details of the datasets used in our experiments.

	MoNuSeg	CPM-17	CoNSeP
Training set	30	32	27
Testing set	14	32	14
Image size	1000×1000×3	500×500×3 or 600×600×3	1000×1000×3
Tissue from Organs	breast, liver, kidney, prostate, bladder, colon, stomach, lung, brain	fat, lymphocytes, blood, vessels, alveoli, red blood cells, normal stroma, cartilage, necrosis	stroma, glands, muscle, collagen, fat
Stain	H&E	H&E	H&E
Patch size	256×256×3	256×256×3	256×256×3

The MoNuSeg dataset provided by Kumar et al. [34] contains 30 images in the training set while 14 images are in the testing set and the resolution of each image is 1000×1000. After applying the color normalization method of Vahadane et al. [35], 16 patches with the size of 256×256 from each image are extracted in the training data with overlapping and 16 patches with the size of 256×256 are extracted from each image in the testing data reshaped into 1024×1024.

The CPM-17 [36] dataset offered by the MICCAI 2017 Digital Pathology Challenge contains 64 images and the resolution of some images is 500×500 while the others are 600×600. To be fair, the dataset split of the training and test sets is the same as the existing methods. The same color normalization method as the MoNuSeg dataset is applied. The training set and the testing set both include 32 images while each image in the dataset is sliced into 4 patches with the size of 256×256 after reshaping to the size of 512×512.

The CoNSeP dataset first appears in HoVer-Net [37] which contains 41 images with the resolution of 1000×1000, and the dataset is collected under a 40× scanner. The dataset split of the training and test sets is also the same as the existing methods. There are 27 images in the training set while 14 images are in the test set, using the same color normalization and image slicing methods as the MoNuSeg dataset.

### Implementation details

Our methods are implemented using Pytorch version 1.10.0 on a workstation equipped with an NVIDIA GeForce RTX 2060, while the machine is running Windows 10. Adam optimizer is applied with a batch size of 4 and the learning rates are set as 1e-4 in U-Net and Ours. The max training epoch is 50 and the validation sets are used to pick the best model during training.

On the training sets of the multiple datasets, about 1/5 samples are randomly selected to form the validation sets. The best model would be saved when the segmentation performance of the network in validate epoch exceeds the previous best before the total number of iterations reaches 50 to evaluate our model’s generalization ability on the test sets.

## Experimental results

To study the generalization performance of our model, several experiments are designed. Firstly, to verify the advance of the structure, comparative experiments among our model and several previous best methods are conducted on the multiple datasets. Secondly, the outcome metrics between semi-supervised and full supervision are compared to verify the effect of the semi-supervised strategy. Finally, to analyze the performance of each part of the structure, ablation experiments are performed to analyze the importance of the edge feature, MEFFA-Block, and shortcut. The ablation studies are conducted on the MoNuSeg dataset because it is representative and the model behaves the same on the multiple datasets.

In this section, the qualitative and quantitative results of our experiments are shown, explaining the reliability of the model structure and the effectiveness of the semi-supervised method.

**Fig 5:**
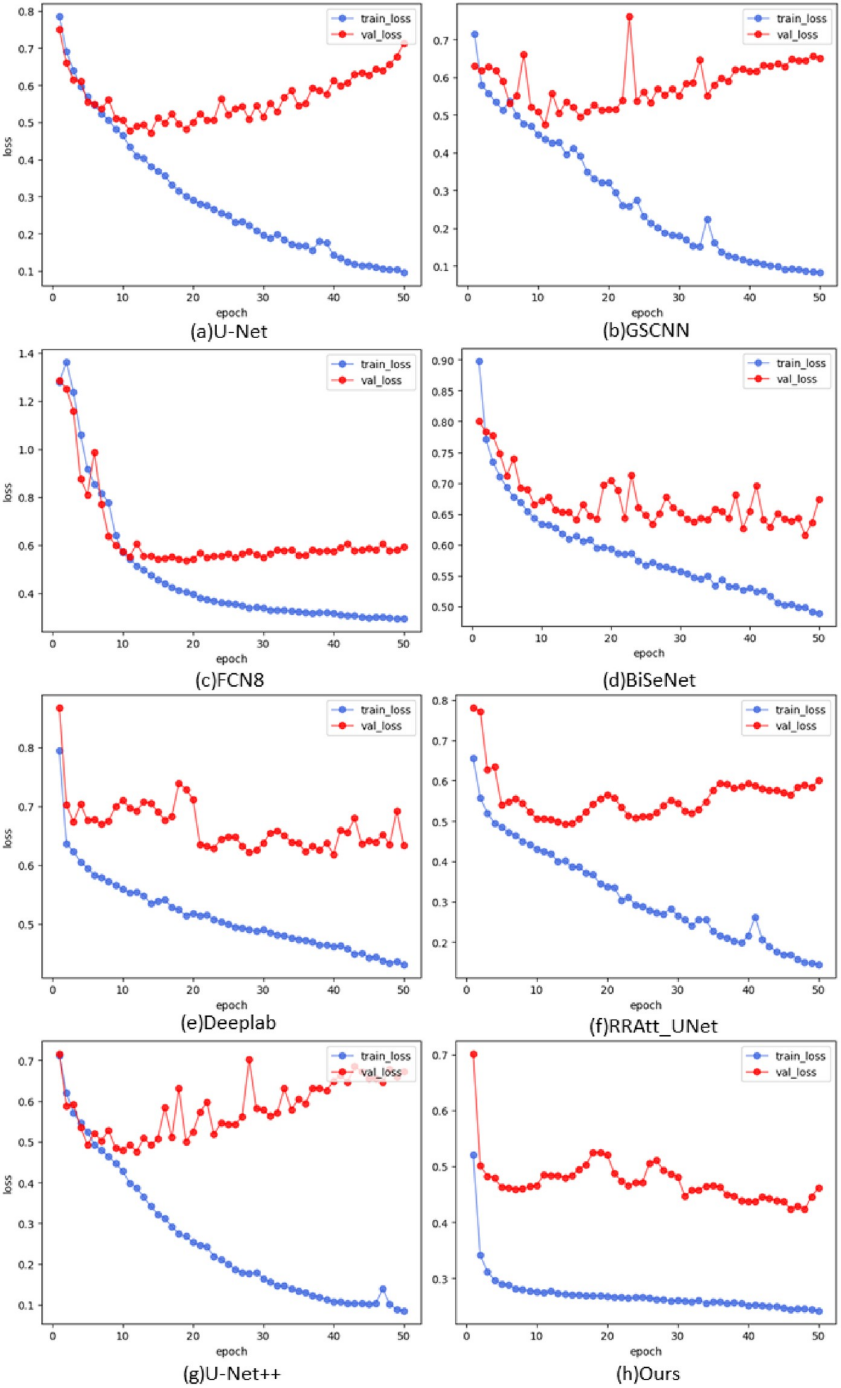
The training and validation graphs on the MoNuSeg dataset.

As shown in Fig 5, we show the training and validation graphs on the MoNuSeg dataset. The generalization abilities of most models have apparent limitations, and their validation set losses are difficult to be lower than 0.5, which will lead to over-fitting when continuing to train the model. Our model is further optimized on the basis of U-Net, which further reduces the loss and produces better segmentation masks.

**Fig 6:**
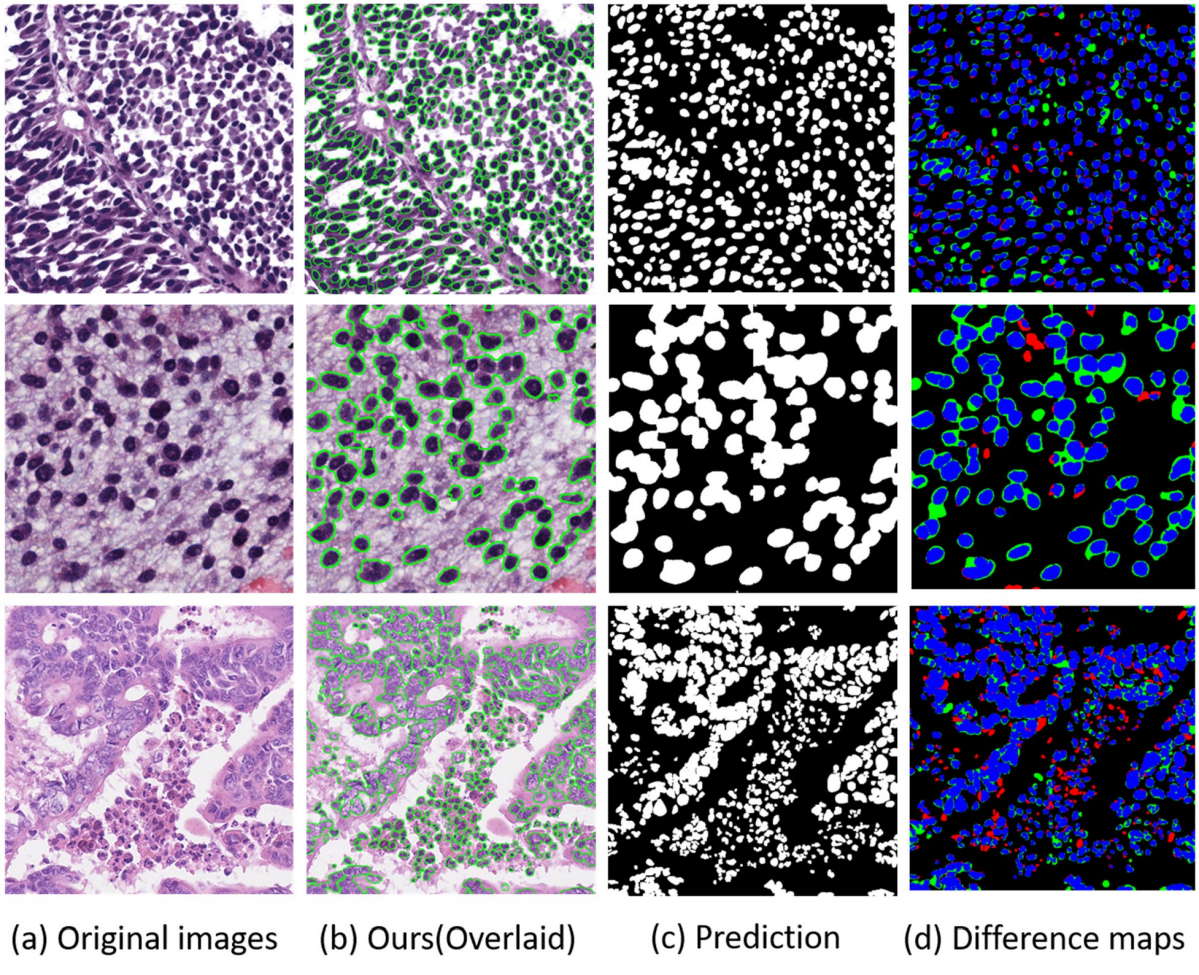
Qualitative results of our models with 100% training samples. The first row is from MoNuSeg, the second row is from CPM-17 and the last row is from CoNSeP. In the Ours(Overlaid) (b), the overlay based on our prediction is shown. In Prediction (c), the output of the MEFFA-Net is shown. In the difference maps (d), referring to [38], blue, green and red areas indicate true positive, false positive and false negative segmentation, respectively.

Fig 6 shows the difference between our model results and ground-truth annotations. The prediction and different maps are shown together, and it can be seen that our method has got accurate segmentation results, with only a small part of under- over-segmentations.

### Comparison to state-of-the-art methods

To verify the superiority of the model structure, comparative experiments with state-of-the-art are designed. Our model is compared with a series of the previous best segmentation methods, including (1) U-Net(2015) [16], (2) FCN8(2015) [39], (3) DeeplabV3+(2018) [40], (4) U-Net++(2018) [41], (5) GSCNN(2019) [42], (6) BiSeNet (2018) [43] and (7) R2Att-UNet(2021) [44]. Four commonly used semantic segmentation evaluation metrics are applied in the experiments for the performance evaluation, including accuracy, SP, IoU and Dice. The same settings in 3.2 are used to train the state of the art and the performance of those methods are gained to compare with ours.

To study the superiority of the model performance, comparative experiments between state of the art and our method are carried out on the multiple datasets.

Working on the multiple datasets, typical metrics of several previous segmentation methods are shown in Table 4. For our algorithm on the MoNuSeg dataset, Dice reaches 0.826, which is better than the previous best GSCNN 0.816 and R2Att-UNet 0.797. IoU reached 0.706, which is not much different from GSCNN’s 0.694. Overall, the proposed model has a well-performance on the MoNuSeg dataset, which has outperformed in comparison with several famous segmentation methods. The performance of our method validates the idea that a better model can be trained when more related features are effectively considered.

**Table 4:** Comparison experimental results on the multiple datasets.

Datasets		MoNuSeg				CPM-17				CoNSeP			
Metrics	Total params	Acc	SP	IoU	Dice	Acc	SP	IoU	Dice	Acc	SP	IoU	Dice
U-Net(2015)	34.53M	0.870	0.844	0.652	0.784	**0.923**	0.926	0.706	0.822	**0.916**	0.923	0.726	0.839
FCN8(2015)	134.27M	0.881	0.891	0.652	0.788	0.892	0.878	0.654	0.788	0.886	0.912	0.647	0.785
DeeplabV3+(2018)	54.70M	0.883	0.883	0.662	0.793	0.918	0.943	0.686	0.809	0.893	0.922	0.655	0.789
U-Net++(2018)	36.63M	0.865	0.838	0.644	0.777	**0.934**	0.952	0.738	0.846	**0.917**	0.922	**0.733**	**0.844**
GSCNN(2019)	137.27M	0.896	0.893	0.694	0.816	**0.935**	0.955	0.741	0.848	0.896	0.899	0.682	0.808
BiSeNet (2018)	12.04M	0.866	0.860	0.633	0.772	0.910	0.942	0.657	0.790	0.889	0.918	0.651	0.788
RRAtt-UNet(2021)	39.44M	0.878	0.854	0.668	0.797	**0.936**	**0.961**	0.742	0.849	**0.917**	**0.957**	0.712	0.831
Ours	40.05M	**0.905**	**0.916**	**0.706**	**0.826**	0.917	0.947	**0.751**	**0.856**	**0.915**	0.922	0.722	0.837

To further verify the generalization performance of the model, more experiments are done on the CPM-17 dataset. For our method on the CPM-17 dataset, the IoU reaches 0.751, which is more than that of classic U-Net++ 0.738, GSCNN 0.741, and R2Att-UNet 0.742. the other segmentation index of our algorithm is also better than the previous best U-Net++ 0.846, GSCNN 0.848 and RRAtt-UNet 0.849. The result indicates that MEFFA-Net could effectively improve the segmentation effect of pathological images nuclei segmentation and be out of the ordinary among many previous methods.

However, such a promotion would not be endless. When U-Net has gained a super result, which usually happens on a simple dataset, our model’s limitation surfaced. As exhibited in the comparison of the CoNSeP dataset, when U-Net has reached Dice of 0.839 and IoU of 0.726, our method just has the similar effect which cannot further improve the segmentation ability of the model.

### Comparison to fully supervised methods

To further study the relationship between the proportion of annotated samples and model performance, comparison experiments among different proportions of the annotated samples are conducted on the multiple datasets.

**Fig 7:**
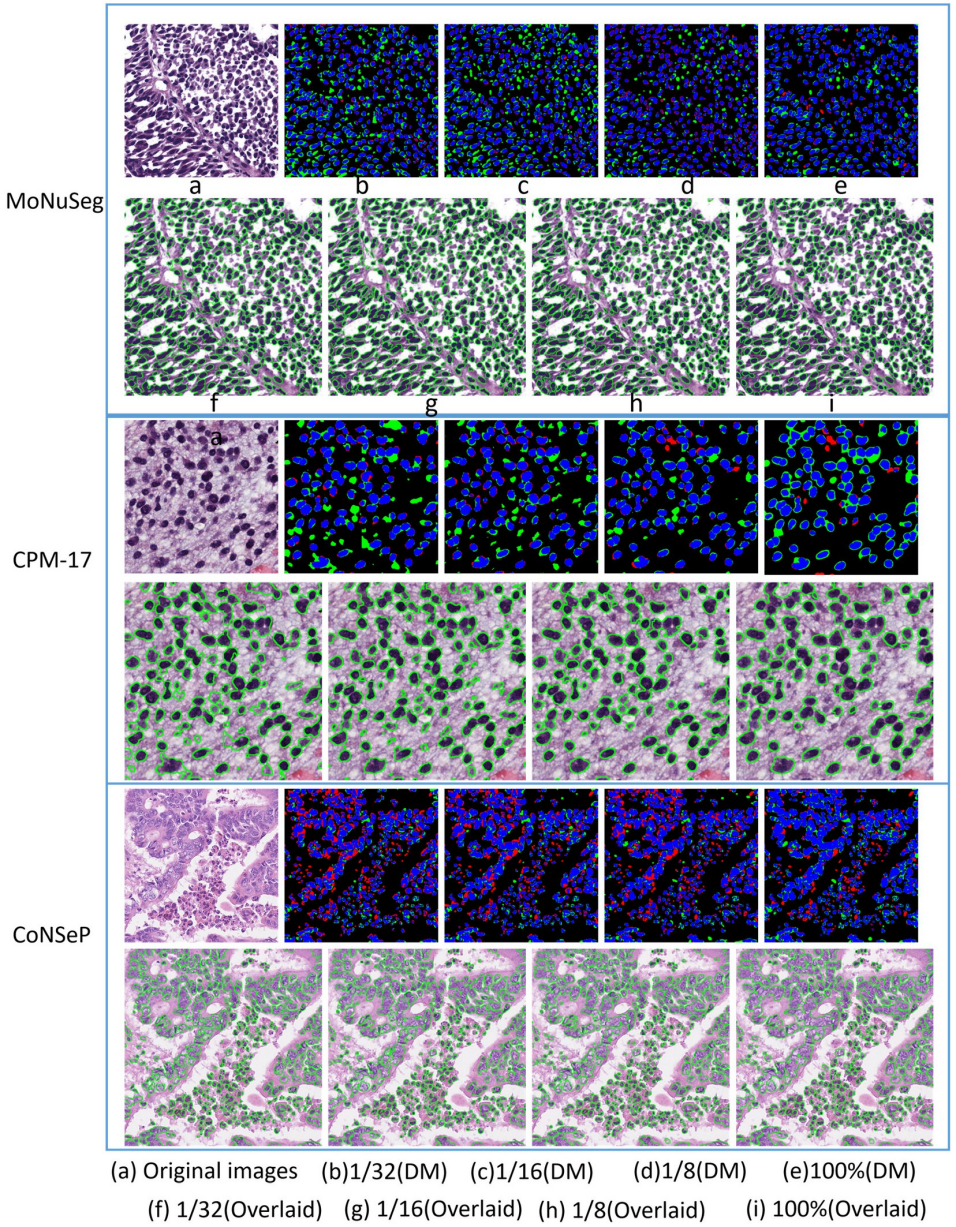
Qualitative comparisons of data dependency. The first, second and the last row are from the MoNuSeg dataset, the CPM-17 dataset and the CoNSeP dataset, respectively. On each row, element (a) is the Original image, while elements (b), (c), (d), and (e) are difference maps of semi-supervised and full-supervised methods which is referring to [38]. The blue, green and red areas indicate true positive, false positive and false negative segmentation, respectively. Then, elements (f), (g), (h), and (i) are the segmentation result of semi-supervised and full-supervised methods.

As shown in Fig 7, accurate segmentation results can be obtained by training with 1/32 labeled samples, while it is still accompanied with under- over-segmentations. With the increase in the proportion of labeled samples, the segmentation accuracy is gradually improved, and under- over-segmentations are alleviated.

As shown in Table 5, comparing with the fully supervised method, conclusions can be derived from experimental results. The MEFFA Net can achieve considerable metrics using only 1/32 of the labeled data. The more proportion of labeled data used, the better the model performance can reach. The performance of MEFFA-Net indicates that our semi-supervised methods are effective in nuclei segmentation and the dependence of supervised learning on a large number of labeled samples is alleviated.

**Table 5:** Comparison experimental results on the multiple datasets.

Datasets	MoNuSeg				CPM-17				CoNSeP			
Proportion	Acc	SP	IoU	Dice	Acc	SP	IoU	Dice	Acc	SP	IoU	Dice
1/32	0.837	0.837	0.573	0.723	0.876	0.868	0.605	0.734	0.864	0.884	0.585	0.725
1/16	0.853	0.849	0.603	0.748	0.895	0.900	0.634	0.768	0.879	0.896	0.615	0.750
1/8	0.874	0.896	0.628	0.767	0.905	0.897	0.674	0.803	0.896	0.925	0.652	0.781
100%	**0.905**	**0.916**	**0.706**	**0.826**	**0.917**	**0.947**	**0.751**	**0.856**	**0.915**	**0.922**	**0.722**	**0.837**

### Ablation study on the MoNuSeg dataset

Due to the same image type and similar model performance on the multiple datasets, MoNuSeg dataset is chosen as a representative dataset for ablation studies, and the results show that our model is well designed.

To verify the effectiveness of the improvement and optimization of our model, the ablation experiments including three aspects are designed to respectively check the effect of edge-aware, MEFFA-Block, and shortcut structure. Experiments on the MoNuSeg dataset show that even without edge-aware, MEFFA-Block, or shortcut structure, the models improve the segmentation results of U-Net. Our methods reach the best performance when all the structures are completed, effectively show that all parts of our method complement each other and greatly promote the segmentation effect under the interaction. Table 6 shows the respective experimental results based on multiple model structures, with the best results for our structure, which indicates the reliability of our model structure.

**Table 6:** Ablation studies results on the MoNuSeg dataset.

Only U-Net	Edge feature	MEFFA-Block	Shortcut	Accuracy	SP	IoU	Dice
✓				0.870	0.844	0.652	0.784
		✓	✓	0.891	0.888	0.685	0.811
	✓		✓	0.900	0.914	0.693	0.817
	✓	✓		0.898	**0.919**	0.686	0.812
	✓	✓	✓	**0.905**	**0.916**	**0.706**	**0.826**

### Detailed display of experimental results

To further visually demonstrate the performance of our model, some segmentation details are shown, illustrating that more accurate segmentation boundaries are obtained by our model.

**Fig 8:**
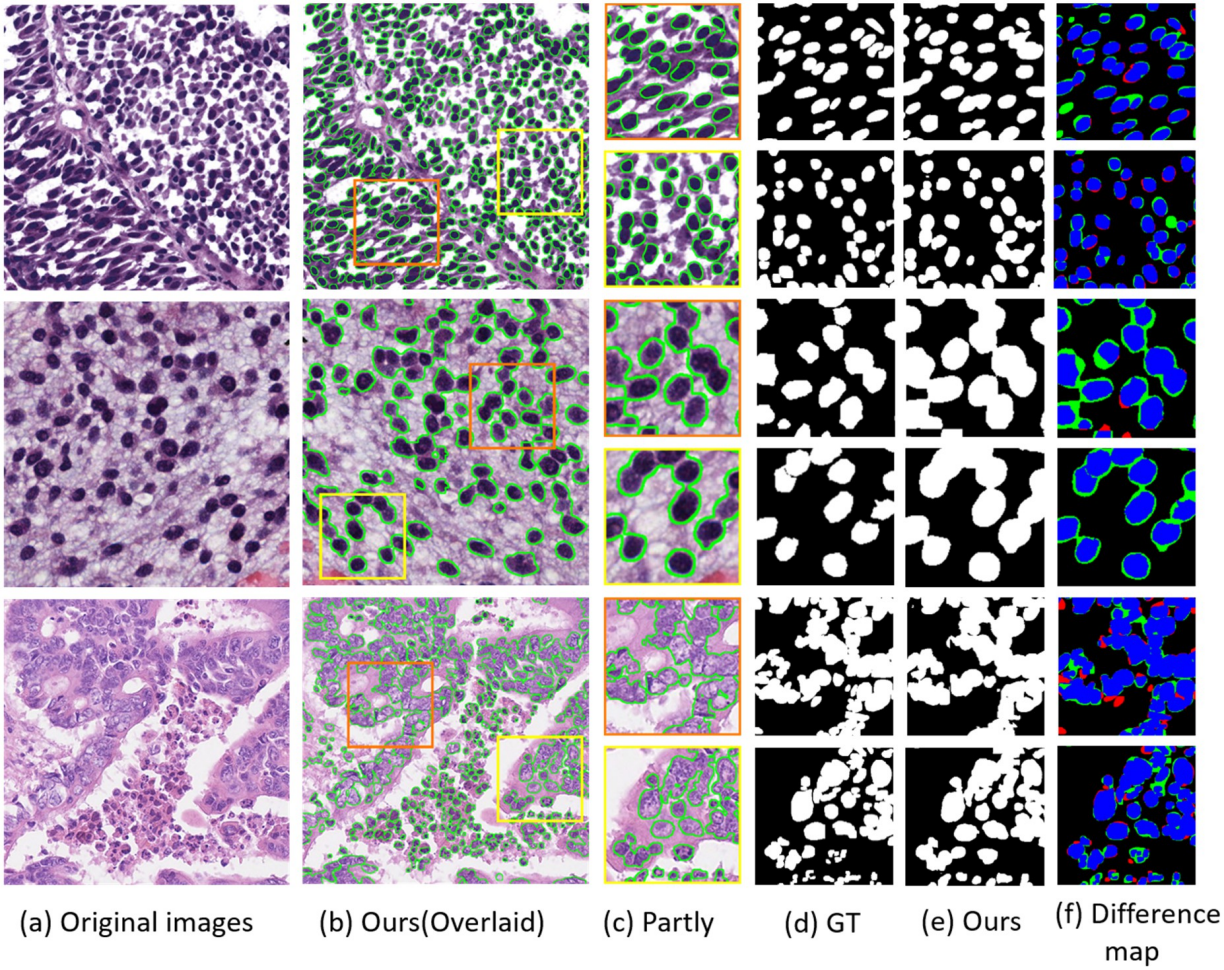
Overlaid of training sample and details of segmentation results. The first, second and last rows are from the MoNuSeg, CPM-17 and CoNSeP dataset, respectively. The (d) and (e) are the ground truth and segmentation results of Ours. In the difference map (f), blue, green and red areas indicate true positive, false positive and false negative segmentation, respectively.

Fig 8 shows the detailed segmentation results of MEFFA-Net on multiple datasets. Our method has made progress in nuclei segmentation of pathological images, and precise segmentation boundaries can be created by our model. With the accurate segmentation boundaries, the basis of pathological diagnosis is more reliable, and it also lays the foundation for further realization of computer-aided diagnosis. Nevertheless, local under- over-segmentations still exist which need to be improved in the future.

## Conclusions

Pathologists mainly determine whether a tumor is benign or malignant based on the cell nucleus’s shape, size, nuclear margin, and nucleus-to-cytoplasm ratio. However, time-consuming and professional manual work urgently requires computer-aided diagnosis methods to improve the efficiency of pathological diagnosis. The first step is to outline cell nuclei from pathology images automatically. Pathological images are difficult to be segmented automatically due to possible huge differences between types, shapes, and staining environments. Moreover, the high cost of manual annotation also makes the development of big data-driven supervised learning methods slow. An automated pipeline based on the MEFFA-Net with pseudo-mask and edge awareness is put forward to create precise nuclei segmentation in pathological images. The MEFFA-Net evolves from the backbone network of Recursive Residual U-Net, taking image, pseudo-mask and edge as inputs while segmentation result and edge detection as outputs. Meanwhile, the classic encoder-decoder structure is one-way, and only the features of the last layer of the encoder can be passed to the decoder which makes it hard to perform global feature processing. The attention mechanism is beneficial in feature transfer and selection. Therefore, MEFFA-Block is designed to lead the network to learn the basic function of nuclei segmentation, but it still has the risking of overfitting and difficult training. Learning from the experience of the residual networks, shortcuts between the pseudo-mask, edge and outputs are added to drive the network to learn the difference between the pseudo-mask and the ground truth, making the MEFFA-Net work well.

Meanwhile, the semi-supervised pipeline is further raised to alleviate the problem of insufficient labeled training samples in a novel way. U-Net is applied to the classic semi-supervised teacher-student model, and original coarse pseudo-masks are obtained. A MEFFA-Net is trained using a few labeled samples, and it is used to enhance the pseudo-masks. Then, seeing the enhanced pseudo-masks as segmentation targets, all the data is used to train a better MEFFA-Net which takes charge of the practical application. The pseudo-mask augmentation strategy based on MEFFA-Net reduces the dependence on manually labeled samples while ensuring considerable accuracy.

The experiment is conducted on three public datasets of MoNuSeg, CPM-17 and CoNSeP, while the division of the datasets refers to the existing methods. The experimental results on the multiple datasets prove the reliability of our method. Surpassing or not inferior to state of the art, the optimized method improves the IoU to 0.706 and 0.751 on the MoNuSeg and CPM-17 datasets, respectively. In the semi-supervised experiment, only using 1/32 of the labels for training can achieve a considerable effect, and using 1/8 of the labels can exceed some mainstream models, which shows the effectiveness of the semi-supervised pseudo-mask augmentation strategy. Ablation studies investigating the importance of edge features, MEFFA-Block, and shortcuts show that our model designing is reasonable.

## Discussions

In this paper, a pathological image nuclei segmentation method based on MEFFA-Net is proposed, which integrates multiple edge features and makes progress in nuclei segmentation. However, there are still some aspects that could be further improved. First, although MEFFA-Net can obtain accurate segmentation boundaries, the phenomenon of under- over-segmentation still exists and the model needs further improvement. Therefore, some post-processing methods such as watershed algorithm can be tried after the model output. Second, on simple datasets, such as the CoNSeP dataset, while U-Net has achieved great results, the MEFFA-Net can’t further improve the accuracy of the segmentation. There may be a bottleneck in the accuracy of nuclei segmentation in pathological images that is difficult to break through. In this regard, we do not need to require completely accurate segmentation, but consider the reliable results of pathological grading and diagnosis under sufficient segmentation accuracy. Third, when designing the loss function, the weights of different losses are roughly determined by a parameter search algorithm and more reliable and effective parameter adjustment methods will be explored in the future. In addition, more useful losses can be designed according to the actual task to guide the training of more practical performance models. Furthermore, the current study is mainly focusing on the precise segmentation of nucleus boundaries, and the whole segmentation of cytoplasm and extracellular matrix has been discussed in our previous work [45]. Precise segmentation of the whole cell body will be studied in the near future.

## Supporting information

S1 Fig Original pathological images of Figs 1, 3, 4, 6–8 in the main body. (ZIP)
