# Supplementary figures and images for: Semi-supervised nuclei segmentation based on multi-edge features fusion attention network
Source: PLoS One. 2023 May 25;18(5):e0286161. doi: 10.1371/journal.pone.0286161 (PMC10212084; doi:10.1371/journal.pone.0286161)

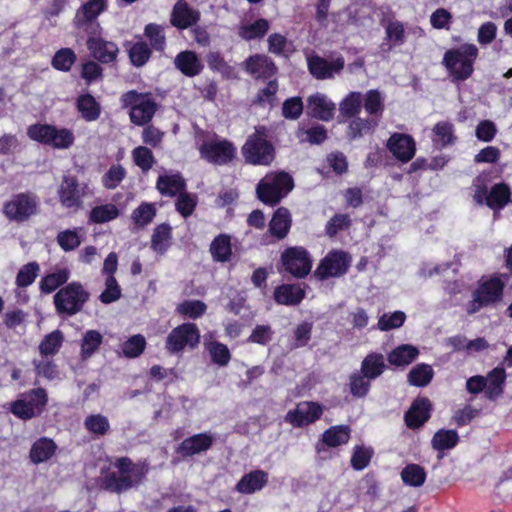

Supplement: S1 Fig — (ZIP) [file pone.0286161.s001.zip › S1/image_25.png]

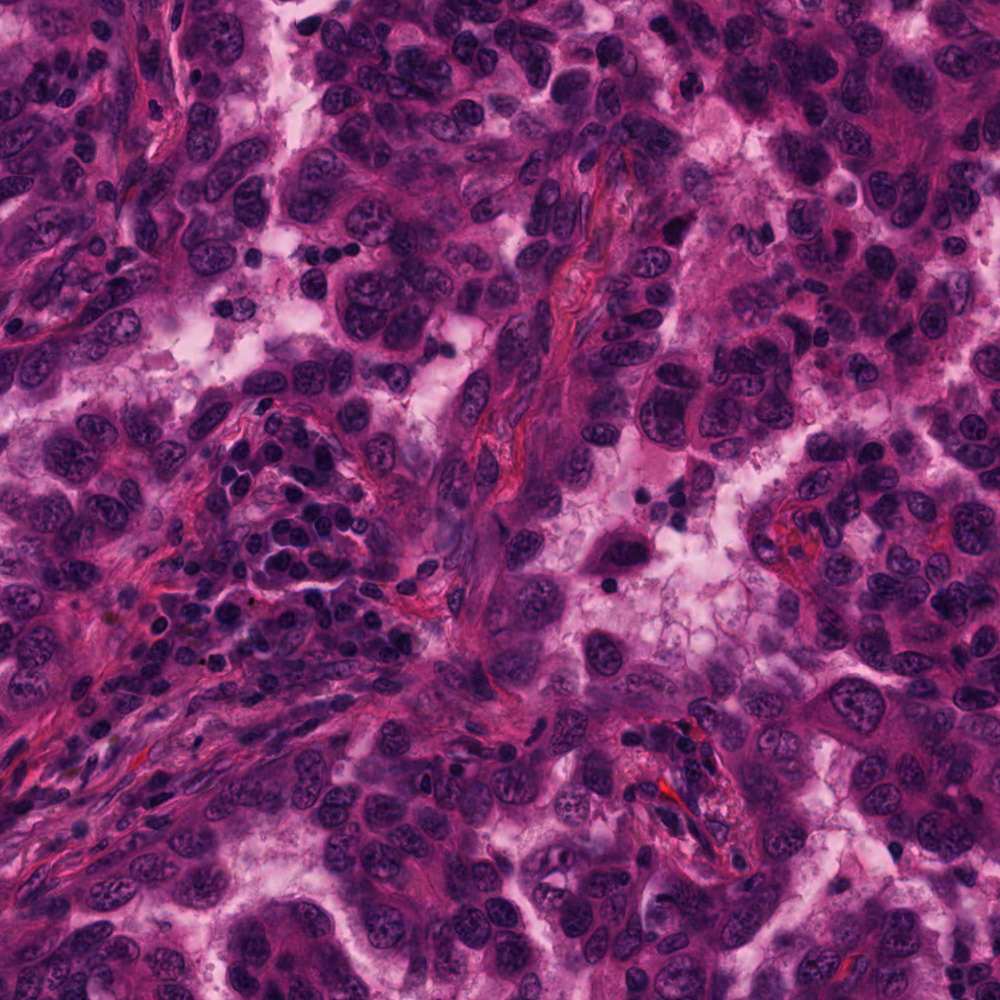

Supplement: S1 Fig — (ZIP) [file pone.0286161.s001.zip › S1/TCGA-49-4488-01Z-00-DX1.tif]

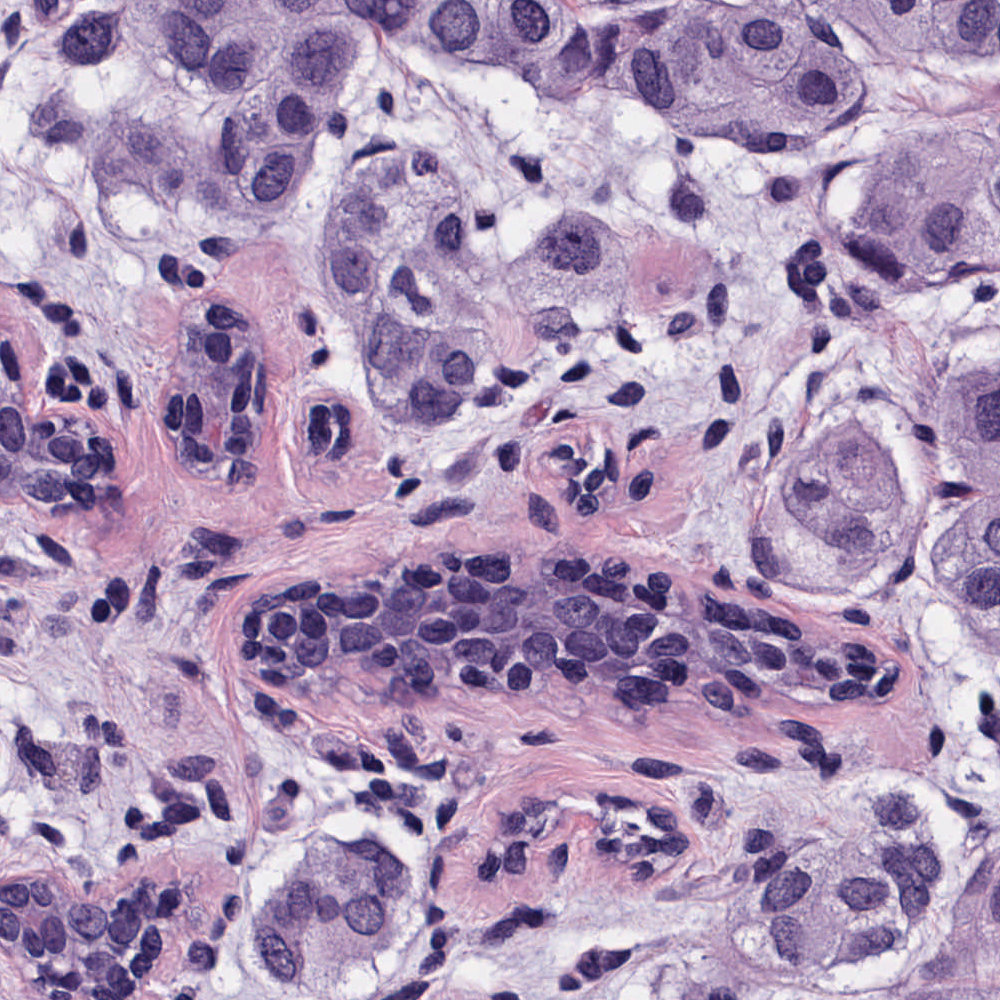

Supplement: S1 Fig — (ZIP) [file pone.0286161.s001.zip › S1/TCGA-A7-A13F-01Z-00-DX1.tif]

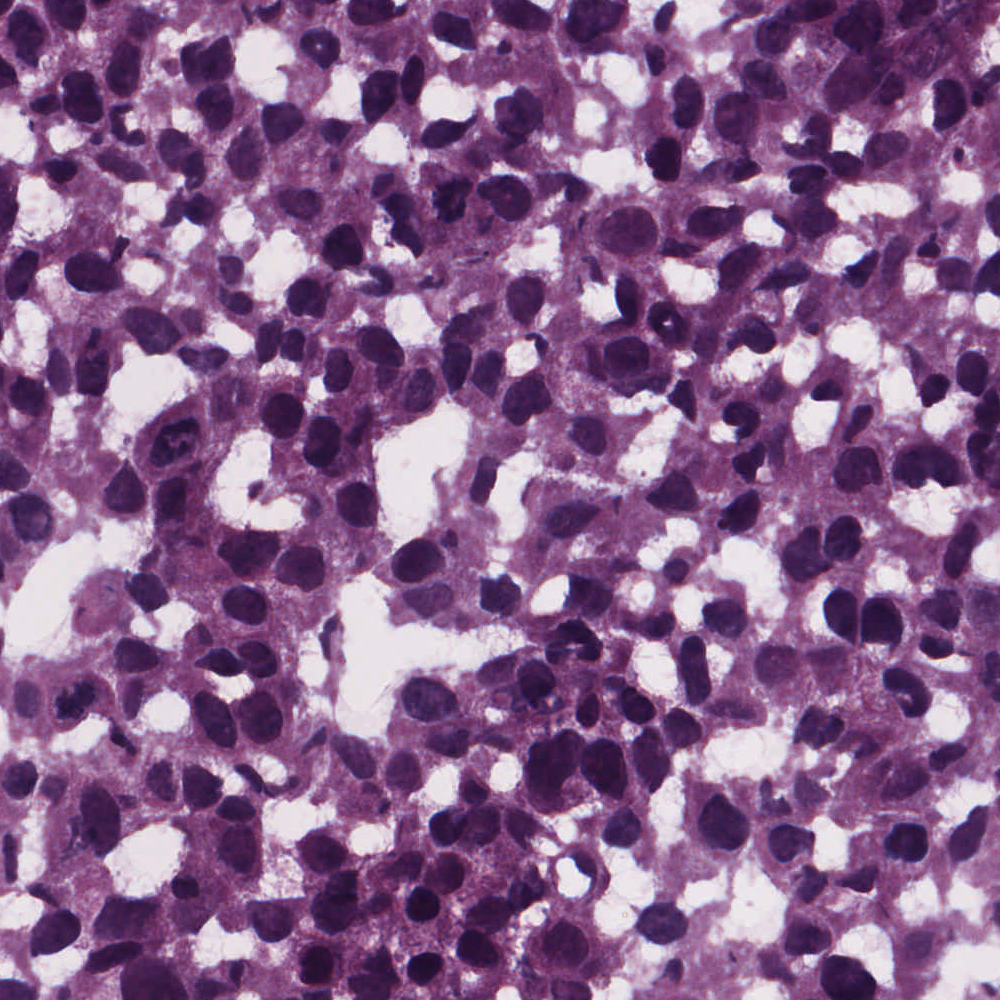

Supplement: S1 Fig — (ZIP) [file pone.0286161.s001.zip › S1/TCGA-DK-A2I6-01A-01-TS1.tif]

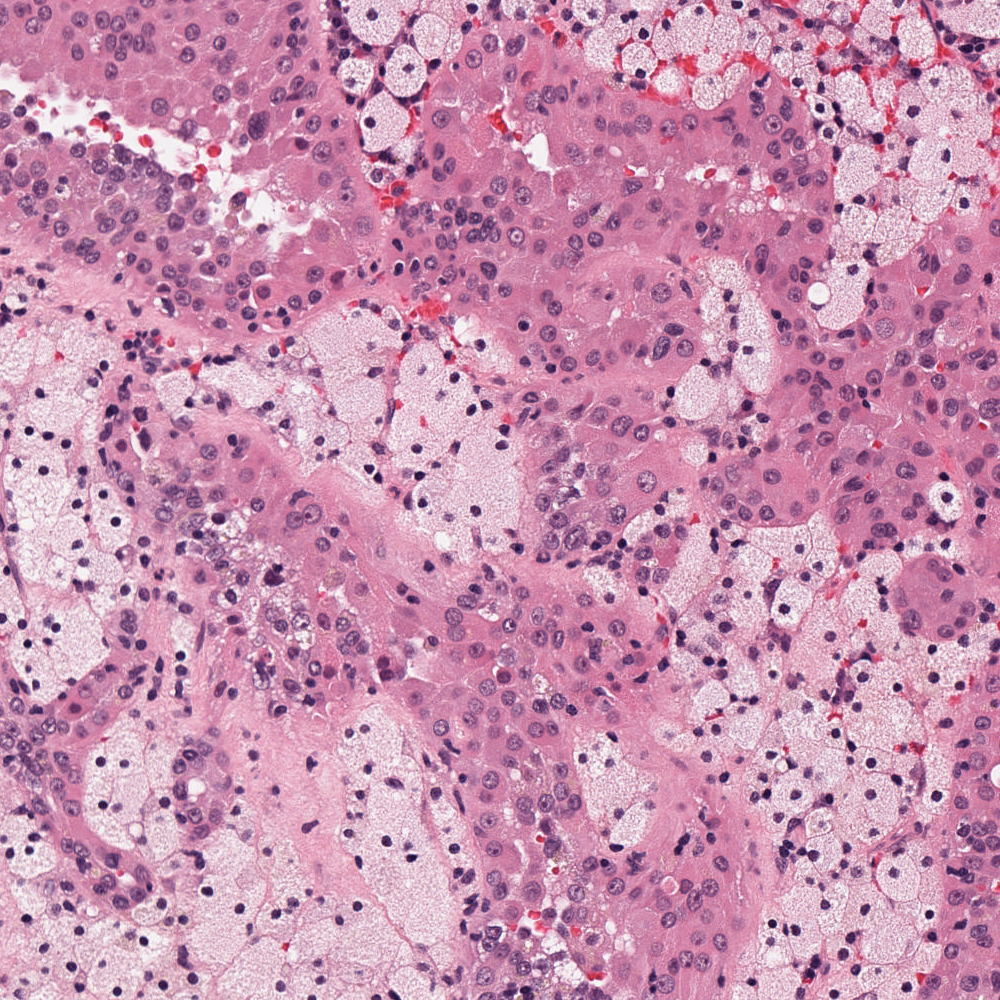

Supplement: S1 Fig — (ZIP) [file pone.0286161.s001.zip › S1/TCGA-HE-7128-01Z-00-DX1.tif]

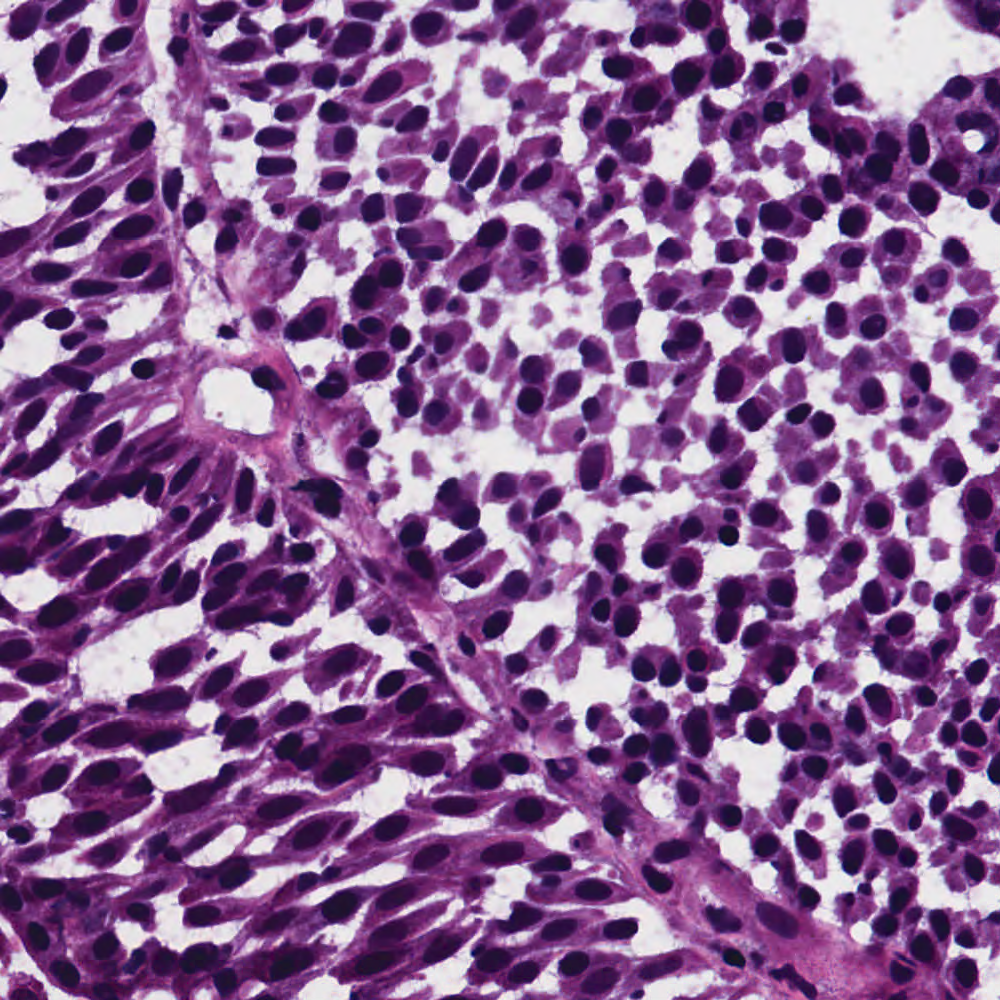

Supplement: S1 Fig — (ZIP) [file pone.0286161.s001.zip › S1/TCGA-ZF-A9R5-01A-01-TS1.tif]

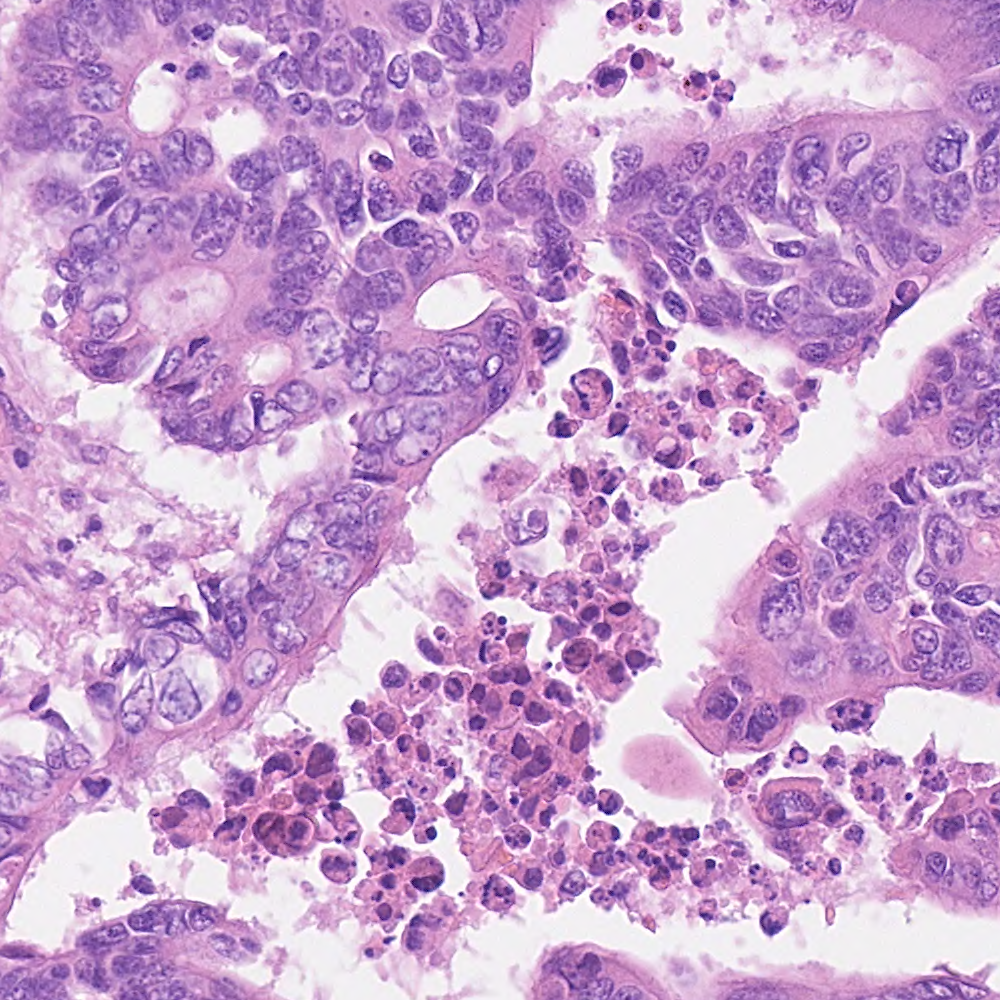

Supplement: S1 Fig — (ZIP) [file pone.0286161.s001.zip › S1/test_11.png]
